# Chronic Migraine and Medication Overuse Headache Worsening After OnabotulinumtoxinA Withdrawn Due to the Severe Acute Respiratory Syndrome-Coronavirus-2 Pandemic

**DOI:** 10.3389/fneur.2021.647995

**Published:** 2021-04-15

**Authors:** Carlo Baraldi, Raffaele Ornello, Valentina Favoni, Simona Sacco, Valeria Caponnetto, Giulia Pierangeli, Luca Pani, Sabina Cevoli, Simona Guerzoni

**Affiliations:** ^1^Department of Biomedical, Metabolic and Neural Sciences, PhD School in Neurosciences, University of Modena and Reggio Emilia, Modena, Italy; ^2^Neuroscience Section, Department of Applied Clinical Sciences and Biotechnology, University of L'Aquila, L'Aquila, Italy; ^3^IRCCS Istituto delle Scienze Neurologiche di Bologna, Bologna, Italy; ^4^Department of Biomedical, Metabolic and Neural Sciences, Medical Toxicology-Headache and Drug Abuse Research Center, University of Modena and Reggio Emilia, Modena, Italy; ^5^Pharmacology Unit, Department of Biomedical, Metabolic and Neural Sciences, University of Modena and Reggio Emilia, Modena, Italy; ^6^Department of Psychiatry and Behavioral Sciences, University of Miami, Miami, FL, United States; ^7^VeraSci, Durham, NC, United States

**Keywords:** chronic migraine, severe acute respiratory syndrome coronavirus-2 pandemic, medication overuse headache, onabotulinumtoxinA, migraine frequency

## Abstract

**Introduction:** OnabotulinumtoxinA (BT-A) is a preventive treatment for chronic migraine (CM), which needs to be administered regularly by a trained clinician every 3 months. The spread of the severe acute respiratory syndrome coronavirus-2 pandemic has forced many patients to momentarily stop the scheduled BT-A injections. The goal of this study was to explore whether those patients experienced a worsening of their CM and, if any, the clinical predictors of migraine worsening after BT-A withdrawal.

**Methods:** This was a retrospective, multicenter study. Patients' clinical data were obtained from their clinical documentation stored at each center. In particular, the following variables were collected: the mean number of headache days in the last month (NHD), the average number of painkillers taken in the last month (AC), the average number of days in which patients took, at least, one painkiller in the last month (NDM), the average intensity of migraine using the numeric rating scale (NRS) score in the last month, and the average score obtained at the six-item Headache Impact Test. The variables mentioned earlier were compared before and after BT-A withdrawal.

**Results:** After BT-A suspension, there was a significant increase in the NHD (*P* = 0.0313, Kruskal–Wallis rank test), AC (*P* = 0.0421, Kruskal–Wallis rank test), NDM (*P* = 0.0394, paired *t*-test), NRS score (*P* = 0.0069, Kruskal–Wallis rank test), and six-item Headache Impact Test score (*P* = 0.0372, Kruskal–Wallis rank test). Patients who were not assuming other preventive treatments other than BT-A displayed similar results. Patients who experienced a >30% worsening in NHD after BT-A was withdrawn displayed a longer CM history (*P* = 0.001, Kruskal–Wallis rank test), a longer MOH duration (*P* = 0.0017, Kruskal–Wallis rank test), a higher AC value at the baseline (*P* = 0.0149, Kruskal–Wallis rank test), a higher NDM (*P* = 0.0024, *t*-test), and a higher average value of the NRS score (*P* = 0.0073, Kruskal–Wallis rank test).

**Conclusion:** BT-A withdrawn during severe acute respiratory syndrome coronavirus-2 pandemic was associated with a general worsening in patients suffering from CM, hence the need to continue BT-A injection to avoid patients' worsening.

## Introduction

Migraine is a primary headache characterized by recurrent attacks of unilateral, pulsating, moderate to severe headache, lasting even for 3 days and associated with nausea, vomiting, photophobia, and/or phonophobia (1). According to the International Classification of Headache Disorders, Third Edition, the recurrence of migraine attacks for ≥8 days per month, in a patient with a total amount of ≥15 headache days per month, for at least 3 months, defines chronic migraine (CM) (1). CM sufferers are often forced to frequently take painkillers to treat recurrent headache attacks, thus developing a secondary headache called medication overuse headache (MOH) (1). CM associated with MOH affects approximately 1–2% of the general population and represents a great challenge for clinicians dealing with headaches both because of the severe patients' impairment and its refractoriness to preventive treatments (2). Indeed, the effectiveness of migraine preventive treatments is lowered by MOH, and a painkiller withdrawal should be performed before prescribing a new preventive drug (3). According to the European Headache Federation (EHF) guidelines, the approved treatments for the prevention of CM are topiramate, onabotulinumtoxinA (BT-A), and monoclonal antibodies acting against calcitonin gene-related peptide or its receptor (4). The efficacy and safety of BT-A in CM prevention have been demonstrated in double-blind, randomized placebo-controlled trials, even in MOH sufferers (5). Additionally, those results have been confirmed, both in terms of effectiveness and safety and for long-term treatments, in several open-label studies (6). According to the EHF guidelines, BT-A injections should be administered regularly every 3 months following the phase 3 trial evaluating migraine prophylaxis therapy scheme (7). However, it is not clear whether the effect of BT-A can last more than 3 months (8). Because BT-A is the only preventive treatment for CM that needs a trained clinician to be administered (9), the spread of the severe acute respiratory syndrome-coronavirus-2 (SARS-COV-2) pandemic has forced many patients to momentarily stop the scheduled BT-A injections. This was due to the conversion of many hospital units into ones dedicated to the management of SARS-COV-2-infected patients, with also the personnel working into the headache centers re-allocated. Since this, the goal of this study was to explore if those patients suffering from CM, with or without MOH, had experienced a worsening in their headache. Moreover, this study aimed to assess possible clinical predictors of CM worsening after BT-A withdrawal.

## Materials and Methods

### Patients

This was a retrospective, non-funded, multicenter study conducted in three Italian headache centers: the Medical Toxicology–Headache and Drug Abuse Research Center of the University of Modena and Reggio Emilia, the Headache Center of the Istituti di Ricovero e Cura a Carattere Scientifico Istituto delle Scienze Neurologiche di Bologna, and the Headache Center of the University of L'Aquila. All patients who had stopped BT-A treatment between February and May 2020 due to the SARS-COV-2 pandemic were reviewed. Patients also affected by MOH or assuming other preventive treatments for CM other than BT-A were included, provided their dosage had remained stable during the withdrawal period. Patients who started other preventive treatments during BT-A withdrawal were excluded. After August 1, 2020, patients were contacted by phone to ask for study participation; in case of a positive response, patients signed informed consent for study participation and data publication during a scheduled visit to the centers. This study was approved by the Area Vasta Nord Ethics Committee (protocol number: 831/2020/OSS/AOUMO), by the Bologna Ethics Committee (as an extension of protocol CE 17082), and by the L'Aquila Ethics Committee (protocol number: 0203392/16). The study was conducted in accordance with the latest version of the declaration of Helsinki.

### Procedures

For every enrolled patient, demographic characteristics and headache features before and after BT-A suspension were deducted from the clinical documentation stored at every center. The following demographic variables were collected: age, sex, body mass index, migraine duration, CM duration, medication overuse duration, ongoing painkiller(s) used, and other preventive treatment(s). At every BT-A scheduled visit, headache diaries and the six-item Headache Impact Test (HIT-6) questionnaire were usually collected to evaluate the headache-related impairment of the patients' quality of life (10). The following tools: the mean number of headache days in the last month [mean number of headache days (NHD)], the average number of painkillers taken in the last month [analgesic consumption (AC)], the average number of days in which patients took, at least, one painkiller in the last month [number of days on medication (NDM)], the average intensity of migraine using the numeric rating scale (NRS) score in the last month, and the average score obtained at the HIT-6 questionnaire in the last month were registered. The variables mentioned earlier were deducted from the clinical documentation produced during the visit for the last injection before the BT-A suspension and during the rescheduled one. Additionally, patients were asked if they, or their relatives, suffered from the SARS-COV-2 infection and if they had continued to go to work or had stayed at home during the period of the BT-A suspension.

### Statistical Analysis

Continuous variables were expressed as mean ± standard deviation, apart from the figures, where the standard error was used. Categorical variables were expressed as proportion and percentages or odds and corresponding 95% confidence interval, when appropriate. The Shapiro–Wilk test was applied to the continuous variables to assess their Gaussian distribution. Continuous normally distributed variables were compared using the paired *t*-test; otherwise, the Kruskal–Wallis rank test was used. Categorical variables were compared using the chi-squared test for the homogeneity of odds. NHD values before and after BT-A withdrawal were compared. Additionally, a similar exploratory analysis was performed regarding the AC, NDM, NRS, and HIT-6 scores. The NHD change was then explored among patients who were not assuming other preventive treatment(s) other than the BT-A to detect the sole effect of the BT-A withdrawal. Even in this case, the changes in variables other than NHD were considered as secondary outcomes. After that, patients were categorized as those who experienced a ≥30% migraine worsening in NHD during the BT-A withdrawal and the ones who had not. This cutoff was chosen because it is commonly accepted as a threshold for the BT-A clinical response (11) so that it could be ruled out that the results of the study are unaffected by the natural fluctuations of migraine (12). Baseline values were compared between patients who had experienced a ≥30% worsening of their migraines and the ones who had not. Furthermore, a multiple logistic regression model was built to study what variables were still significantly associated with CM worsening at the multivariate one. The Pearson's χ^2^ goodness of fit test was then performed to assess the overall goodness of fit of the model. A receiver operating characteristic (ROC) curve analysis was performed to detect the optimal cut-point of continuous variables discriminating between patients' who had experienced a migraine worsening during the BT-A withdrawal and the ones who had not. The sample size was not calculated due to the retrospective nature of the study. However, according to the calculations made with G^*^Power software (13), we estimated that performing a *t*-test analysis with a total sample size of *n* = 52 would be sufficient to detect a large (*f* = 0.8) effect size between two groups with a *P*-value < 0.05 and 80% power. *P*-values lower than 0.05 were considered significant. Statistical calculations, apart from the sample size, were made with STATA Ic15 software.

## Results

**Figure 1:**
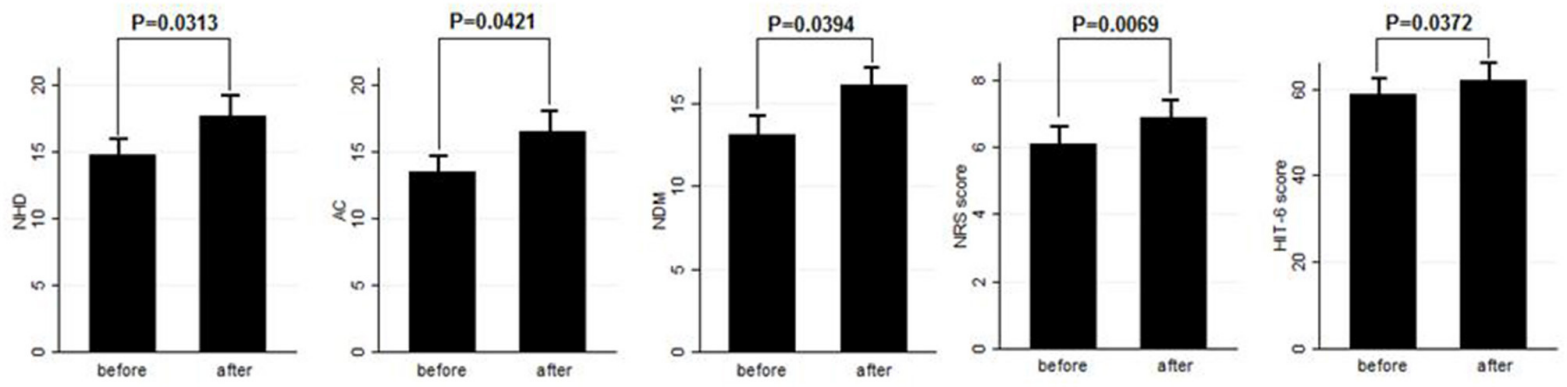
Comparison of NHD, AC, NDM, NRS, and HIT-6 score before and after BT-A withdrawal. Range bars indicate the corresponding standard errors. All comparisons were made with the Kruskal–Wallis rank test, despite for NDM where a paired *t*-test was applied.

**Figure 2:**
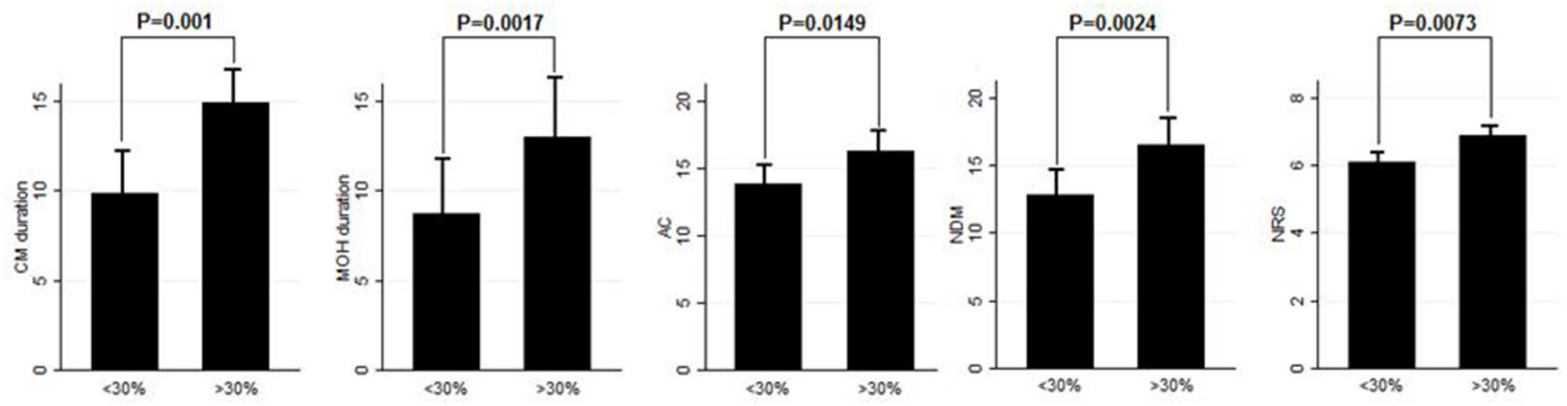
Comparison of CM duration, MOH duration, AC, NDM, and NRS score between patients who worsened by ≥30% in the NHD and those who did not. Range bars indicate the corresponding standard errors. All comparisons were made with the Kruskal–Wallis rank test, despite for NDM were a *t*-test was applied.

Eighty patients were enrolled, mostly females (60/80, 75%). The mean age was 50.41 ± 12.06 years, and the mean body mass index was 24.3 ± 5.34. The analyzed sample had a mean migraine duration of 32.61 ± 13.73 years, whereas the average CM duration was 12.35 ± 12.01 years. Sixty patients (75%) suffered from CM associated with MOH. The medication overuse had lasted for an average period of 11.07 ± 10.67 years. The most frequently overused painkillers were triptans (50/80, 62.5%), followed by non-steroidal anti-inflammatory drugs (23/80–28.75%) and the associations of painkillers (9/80, 11.25%). Thirty-four patients (42.5%) took other preventive medications other than BT-A, most commonly beta-blockers (19/80, 23.75%). The delay of the BT-A treatment was 52.14 ± 26.27 days. Patients delayed the BT-A treatment after a mean of 4 ± 1 injections; 36 patients (45%) had undergone >3 injection cycles before delaying the BT-A administration. These results are summarized in Table 1. After the BT-A suspension, there was a significant increase in the NHD (14.78 ± 7.71 *vs*. 17.35 ± 8.8, *P* = 0.0313, Kruskal-Wallis rank test). Among the explored secondary outcomes, AC (13.53 ± 8.53 *vs*. 16.23 ± 9.94, *P* = 0.0421, Kruskal–Wallis rank test), NDM (13.14 ± 8.03 *vs*. 15.79 ± 9.16, *P* = 0.0394, paired *t*-test), NRS score (6.1 ± 1.92 *vs*. 6.87 ± 1.92, *P* = 0.0069, Kruskal–Wallis rank test), and HIT-6 score (59 ± 8.16 *vs*. 62 ± 6.8, *P* = 0.0372, Kruskal–Wallis rank test) significantly increased after BT-A withdrawn. All these data are summarized in Figure 1. After the BT-A withdrawal, patients who were not taking other preventive treatments for CM displayed a higher NHD (14.59 ± 8.25 *vs*. 18.47 ± 9.45, *P* = 0.0001, paired *t*-test). Regarding the secondary outcomes, a higher NDM (12.62 ± 8.16 *vs*. 16.84 ± 9.71, *P* < 0.0001, paired *t*-test), a higher NRS score (6.62 ± 1.7 *vs*. 7.51 ± 1.58, *P* = 0.0002, paired *t*-test), and a higher HIT-6 score (59.03 ± 8.6 *vs*. 62.31 ± 6.99, *P* = 0.0292, Kruskal–Wallis rank test) were registered among patients taking only BT-A. No significant differences were found regarding the AC (13 ± 7.97 *vs*. 16.89 ± 9.74, *P* = 0.0871, Kruskal–Wallis rank test). These data are summarized in Table 2. Patients who experienced a >30% worsening in NHD after the BT-A withdrawal displayed a longer CM history (14.92 ± 10.04 *vs*. 9.9 ± 13.3, *P* = 0.001, Kruskal–Wallis rank test) and a longer MOH duration (8.74 ± 12.86 *vs*. 13.03 ± 8.11, *P* = 0.0017, Kruskal–Wallis rank test). Moreover, they also had a higher AC value at the baseline (13.84 ± 10.36 *vs*. 16.29 ± 7.91, *P* = 0.0149, Kruskal–Wallis rank test), a higher NDM (12.79 ± 8.95 *vs*. 16.55 ± 7.95, *P* = 0.0024, *t*-test), and a higher average value of NRS score (6.1 ± 1.94 *vs*. 6.88 ± 1.99, *P* = 0.0073, Kruskal–Wallis rank test). No other significant differences were found. These results are summarized in Figure 2. No significant differences were found between patients assuming other preventive treatments and those who were not among those who experienced a ≥30% worsening of NHD (data not shown). The ROC curve analysis revealed that a CM duration of ≥6.5 years predicted a ≥30% migraine worsening after the BT-A withdrawal with a sensitivity of 79% and a specificity of 63%. Moreover, a MOH duration of ≥6.5 years predicted migraine worsening with a sensitivity of 76% and a specificity of 71%. The consumption of ≥13 painkillers per month predicted the migraine worsening with a sensitivity of 71% and a specificity of 54%, and an NRS score higher than 7.5 predicted a migraine worsening with a sensitivity of 55% and a specificity of 71%. All these data are summarized in Table 3. The multiple logistic regression model revealed that AC and MOH duration were significantly associated with the migraine worsening (NDM was dropped-out due to collinearity with AC). The Pearson's χ^2^ goodness of fit test revealed that the model fitted reasonably well the migraine worsening (Pearson's χ^2^ = 65.25, *P* = 0.2393, data not shown).

**Table 1:** Demographic characteristics.

**Variable**	
Number of patients	80/80 (100%)
Female	60/80 (75%)
Age	50.41 ± 12.06
Body mass index	24.3 ± 5.34
Migraine duration	32.61 ± 13.73
CM duration	12.35 ± 12.01
MOH	60/80 (75%)
MOH duration	11.07 ± 10.67
Triptans	50/80 (62.5%)
NSAIDs	23/80 (28.75%)
Associations of painkillers	9/80 (11.25%)
*Other preventive treatments*	35/80 (43.75%)
TCA	10/80 (12.5%)
Beta-blockers	19/80 (23.75%)
Flunarizine	1/80 (1.25%)
Anticonvulsants	7/80 (8.75%)
SSRI-SNRI	12/80 (15%)
*Number of BT-A injection before withdrawn*	4 ± 1
*2*	14/80 (17.5%)
*3*	30/80 (37.5%)
*≥3*	36/80 (45%)
Total days delayed	52.14 ± 26.27
Mean time from the last BT-A injection	142.14 ± 26.27

*CM, chronic migraine; MOH, medication overuse-headache; NSAIDs, non-steroidal anti-inflammatory drugs; TCA, tricyclic antidepressants, BT-A, onabotulinumtoxinA; SSRI, selective serotonin reuptake inhibitor; SNRI, serotonin and norepinephrine reuptake inhibitor*.

**Table 2:** Comparison of NHD, AC, NDM, NRS, and HIT-6 scores before and after BT-A withdrawal in patients who did not take any other preventive medication for CM.

**Variable**	**Before BT-A withdrawal**	**After BT-A withdrawal**	***P*-value**
NHD	14.59 ± 8.25	18.47 ± 9.45	0.0001
AC	13 ± 7.97	16.89 ± 9.74	0.0871
NDM	12.62 ± 8.16	16.84 ± 9.71	<0.0001
NRS score	6.62 ± 1.7	7.51 ± 1.58	0.0002
HIT-6 score	59.03 ± 8.6	62.31 ± 6.99	0.0292

**Table 3:** Optimal cut-points better discriminating patients' who worsened after BT-A stoppage and who did not.

**Variable**	**ROC curve AUC**	**Cut-point**	**Sensitivity**	**Specificity**
CM duration	0.7139	6.5	79%	63%
MOH duration	0.7228	6.5	76%	71%
AC	0.6115	13	71%	54%
NDM	0.6391	16	55%	71%
NRS	0.6237	7.5	49%	71%

## Discussion

This study explored the evolution of CM with and without medication overuse in those patients who were forced to momentarily withdraw the BT-A injections due to the SARS-COV-2 pandemic. After BT-A withdrawal, patients displayed a worsening in migraine frequency. Moreover, exploratory analysis upon analgesic intake, pain intensity, and quality of life revealed a general worsening in all these parameters. Indeed, the NHD, the AC, the NDM, the NRS, and the HIT-6 scores were significantly increased at the time of BT-A restart (Figure 1). In literature, there are no clear indications on whether to stop the BT-A injections: the eventual suspension of BT-A should be based on the evaluation of the number of headache days per month that patients displayed (8), which is why this parameter was chosen as the primary outcome. Anyhow, even in the case of a good response to BT-A, the consequence of its withdrawal may be the relapse of CM and MOH (8), and, moreover, the few studies that explored this issue produced conflicting results. Indeed, Guerzoni and collaborators found a general worsening of the patients' conditions (14), and, similarly, Cernuda-Morollòn and co-workers found out that 90% of patients reaching the year of treatment with the BT-A needed to continue it without delay to keep migraine frequency under control (15). On the contrary, Rothrock's group did not find, after 6 months, a worsening in migraine frequency in the group of “super-responders” to the BT-A, i.e., those patients achieving a migraine frequency and disability level for which prophylaxis is usually not indicated (16). The present study seems to confirm the results achieved by the first two ones (14, 15), likely because of the higher rate of MOH sufferers in the analyzed sample if compared with the one of Rothrock's group (16). MOH increases CM patients' refractoriness to preventive treatments (3), and the analyzed sample was featured by a long CM history and a long MOH duration (Table 1). Since this, the long CM and MOH duration may account for the rapid relapse after BT-A withdrawal. Notably, an increase in NHD was also achieved in those patients who were taking solo the BT-A as preventive treatment (Table 2) so that the observed worsening could be linked to the BT-A suspension. Considering this, patients undergoing the BT-A withdrawal should be strictly monitored to prevent CM and MOH relapse. The gradual increase of the inter-injection period length for 4/5 months should be preferred instead of a sudden interruption to abandon the BT-A treatment (17). Anyhow, even in the case of a sudden interruption, the EHF guidelines recommend the prudent re-evaluation of patients after 4–5 months from the BT-A withdrawal (8). The action of BT-A persists even for 5 months in central neurons *in vivo* (18) but is probably lower than expected in humans, thus justifying the trimestral injection protocol followed in the phase 3 trial evaluating migraine prophylaxis therapy program (19). Regarding this point, it would be interesting to know when CM and MOH sufferers begin to worsen after the BT-A suspension. Despite the lack of a significant association between the length of the BT-A suspension and the response status, the general worsening in the patient's conditions suggests that CM worsening occurs 4 months before the suspension as also intuitable from the BT-A mechanism of action in CM. As the withdrawal of BT-A is associated with the worsening of CM, it might be interesting to evaluate whether some patients' features may predict the worsening of CM and MOH upon the BT-A discontinuation. Patients who displayed a more severe worsening were the most impaired at the baseline: they displayed a higher CM and MOH duration and a higher value of AC, NDM, and NRS scores. The present results are in partial agreement with the ones achieved by Lee and collaborators, who found out that a long CM duration was associated with a poor outcome during the BT-A treatment (20). Additionally, Dominguez and collaborators found that a longer CM duration and higher pain intensity were predictors of a bad response to the BT-A (21, 22), besides being predictors of CM worsening after the BT-A withdrawal, as the present study highlighted. The amount of painkillers taken has never been linked specifically to a poor response to the BT-A, but this study revealed that patients with a higher AC at the baseline were more likely to experience a worsening in their migraine after the BT-A withdrawal (Figure 2). The overuse of painkillers may lower the activation threshold of the trigeminal nerve fibers: Buonvicino and co-workers discovered that the administration of indomethacin and eletriptan in rats for 1 month enhanced the expression of neuropeptides, their receptors, prostanoids, TRPV2, and TRPA1 in the rat trigeminal neurons (23, 24). Moreover, chronic acetaminophen administration had proven to increase the expression of 5-HT2A receptors in the trigeminal ganglion of exposed rats, increasing trigeminal excitability (25). In humans, MOH patients have a higher cutaneous threshold than patients without MOH, suggesting that the prolonged overuse of analgesics may lower the trigeminal threshold (26), leading to trigeminal sensitization (27). Considering this, it is reasonable that patients with higher AC and NDM and a longer MOH duration at the baseline, i.e., the most impaired, may have a higher risk of worsening after the BT-A withdrawal. In particular, from the present study, a MOH history longer than 6 years and a half predicts relapse after the BT-A withdrawal with a sensitivity of 76% and a specificity of 71%. Moreover, the consumption of at least 13 painkillers per month predicts migraine relapse after the BT-A withdrawal with a sensitivity of 71% and a specificity of 54% (Table 3). However, these results should be taken carefully due to the low specificity and sensitivity of the ROC curves mentioned earlier. This study has some limits, such as the lack of a control group, its retrospective nature, and the limited number of enrolled patients. Additionally, the different length of the withdrawal period is a limit, but it was inevitable because of the sudden stop of the BT-A injection during the SARS-COV-2 pandemic. Anyway, the present results are one of the few to explore the CM evolution, with or without MOH, after BT-A withdrawal and gain more importance considering that the spread of the SARS-COV-2 pandemic, which has generally ameliorated the conditions of the migraineurs (28). This is probably due to the avoidance of all those migraine triggers that may precipitate migraine attacks (29). Despite this, the worsening of patients after the forced suspension of the BT-A injection due to the SARS-COV-2 pandemic reinforced the data about its effectiveness and put the question upon suspending or not the BT-A, even during concurrent health emergencies such as pandemics. Indeed, this study highlights that delaying the BT-A treatment may be detrimental for chronic migraineurs complicated with MOH, particularly those with a high burden of long-standing CM and MOH. More studies upon bigger cohorts are needed to clearly define the disadvantages of withdrawing the BT-A in patients suffering from CM and MOH.

## Data Availability Statement

The raw data supporting the conclusions of this article will be made available by the authors, without undue reservation.

## Ethics Statement

The studies involving human participants were reviewed and approved by Area Vasta Emilia Nord ethics committee, Bologna ethics committee and L'Aquila ethics committee. The patients/participants provided their written informed consent to participate in this study.

## Author Contributions

SC, SS, LP, and SG conceived the article. CB, VF, RO, SC, SS, and SG recruited patients. CB, VF, and RO wrote the manuscript. CB and SG made a statistical analysis of data. All authors have read and approved the last version of the manuscript.

## Conflict of Interest

CB received travel grants and honoraries from Allergan, Eli Lilly, Teva, and Novartis. SG received travel grants and honoraries from Allergan, Eli Lilly, Teva, and Novartis. LP is the Chief Scientific Officer of EDRA-LSWR Publishing Company and Inpeco SA Total Lab Automation Company. In the last year, he has been a scientific consultant to AbbVie, USA; BCG, Switzerland; Boehringer-Ingelheim, Germany; Compass Pathways, UK; Johnson & Johnson, USA; Takeda, USA; VeraSci, USA; and Vifor, Switzerland. VF, GP, and SC received speaker honoraria and funding for travel and has received honoraria for participation in advisory boards sponsored by Allergan, Eli Lilly, Novartis, and Teva. RO received funding from Allergan, Novartis, and Teva and personal fees from Novartis and Eli Lilly. SS received fees as speaker or advisor for Abbott, Allergan, AstraZeneca, Eli Lilly, Novartis, and Teva. She received research grants from Allergan and Novartis. She received nonfinancial supports from Abbott, Allergan, Bayer, Bristol-Myers Squibb, Daiichi-Sankyo, Eli Lilly, Medtronic, Novartis, Pfizer, Starmed, and Teva. She received fees for CME/education from Medscape. The remaining author declares that the research was conducted in the absence of any commercial or financial relationships that could be construed as a potential conflict of interest.

## Abbreviations

AC: analgesic consumption
BT-A: onabotulinumtoxinA
CM: chronic migraine
EHF: European Headache Federation
HIT-6: six-items headache impact test
MOH: medication overuse headache
NDM: number of days on medication
NHD: number of headache days
NRS: numeric rating scale
ROC: receiver operating characteristic
SARS-COV-2: severe acute respiratory syndrome-coronavirus-2.
